# Capture-Recapture Among Men Who Have Sex With Men and Among Female Sex Workers in 11 Towns in Uganda

**DOI:** 10.2196/12316

**Published:** 2019-04-03

**Authors:** Kevin Apodaca, Reena Hemendra Doshi, Moses Ogwal, Herbert Kiyingi, George Aluzimbi, Geofrey Musinguzi, Ibrahim Lutalo, Evelyn Akello, Wolfgang Hladik

**Affiliations:** 1 Public Health Institute Oakland, CA United States; 2 Centers for Disease Control and Prevention Center for Global Health Division of Global HIV and TB Atlanta, GA United States; 3 Centers for Disease Control and Prevention Epidemic Intelligence Service Atlanta, GA United States; 4 Makerere University School of Public Health Kampala Uganda; 5 Centers for Disease Control and Prevention Division of Global HIV and TB Kampala Uganda; 6 Makere University School of Public Health Monitoring and Evaluation Techical Support Program Kampala Uganda

**Keywords:** sex worker, population size, men who have sex with men, HIV

## Abstract

**Background:**

Key populations at higher risk for HIV infection, including people who inject drugs, men who have sex with men (MSM), and female sex workers (FSWs), are disproportionately affected by the HIV/AIDS epidemic. Empirical estimates of their population sizes are necessary for HIV program planning and monitoring. Such estimates, however, are lacking for most of Uganda’s urban centers.

**Objective:**

The aim of this study was to estimate the number of FSWs and MSM in select locations in Uganda.

**Methods:**

We utilized conventional 2-source capture-recapture (CRC) to estimate the population of FSWs in Mbale, Jinja, Wakiso, Mbarara, Gulu, Kabarole, Busia, Tororo, Masaka, and Kabale and the population of MSM in Mbale, Jinja, Wakiso, Mbarara, Gulu, Kabarole, and Mukono from June to August 2017. Hand mirrors and key chains were distributed to FSWs and MSM, respectively, by peers during capture 1. A week later, different FSWs and MSM distributors went to the same towns to collect data for the second capture. Population size estimates and 95% CIs were calculated using the CRC Simple Interactive Statistical Analysis.

**Results:**

We estimated the population of FSWs and MSM using 2 different recapture definitions: those who could present the object or identify the object from a set of photos. The most credible (closer to global estimates of MSM; 3%-5%) estimates came from those who presented the objects only. The FSW population in Mbale was estimated to be 693 (95% CI 474-912). For Jinja, Mukono, Busia, and Tororo, we estimated the number of FSWs to be 802 (95% CI 534-1069), 322 (95% CI 300-343), 961 (95% CI 592-1330), and 2872 (95% CI 0-6005), respectively. For Masaka, Mbarara, Kabale, and Wakiso, we estimated the FSWs population to be 512 (95% CI 384-639), 1904 (95% CI 1058-2749), 377 (95% CI 247-506), and 828 (95% CI 502-1152), respectively. For Kabarole and Gulu, we estimated the FSWs population to be 397 (95% CI 325-469) and 1425 (95% CI 893-1958), respectively. MSM estimates were 381 (95% CI 299-462) for Mbale, 1100 (95% CI 351-1849) for Jinja, 368 (95% CI 281-455) for Wakiso, 322 (95% CI 253-390) for Mbarara, 180 (95% CI 170-189) for Gulu, 335 (95% CI 258-412) for Kabarole, and 264 (95% CI 228-301) for Mukono.

**Conclusions:**

The CRC activity was one of the first to be carried out in Uganda to obtain small town–level population sizes for FSWs and MSM. We found that it is feasible to use FSW and MSM peers for this activity, but proper training and standardized data collection tools are essential to minimize bias.

## Introduction

### Background

Key populations such as female sex workers (FSWs) and men who have sex with men (MSM) are disproportionately affected by the HIV epidemic [1,2]. FSWs are estimated to be 13.5 times more likely to become infected with HIV than the general female population, whereas MSM are 19 times more likely to be living with HIV than the general male population [3]. In sub-Saharan Africa, the estimated HIV prevalence among FSWs is 36.9%, whereas it is 17.9% among MSM [4,5]. Sexual risk behavior and number of sexual partners, along with violence, criminalization, and stigma may contribute to their increased HIV vulnerability [6].

Uganda has a generalized epidemic; however, the estimated HIV prevalence among FSWs and MSM (33% [7] and 13.7% [8], respectively) are substantially higher than that in the general population (6.2% [9]). In Uganda, FSWs and MSM face stigma and marginalization, limiting their access to prevention and treatment programs. Thus, targeted HIV services for prevention, care, and treatment need to be specifically planned and evaluated for such populations. Such planning and evaluation requires an accurate estimate of the population of FSWs and MSM.

There is currently no gold standard method on estimating the size of key populations. Several population size estimation (PSE) methods have been applied in different settings, but each method has its own set of potential biases and limitations [10]. Capture-recapture (CRC) has been recommended for use when a census is not feasible and if there are no or poor quality service data, which is the case in Uganda [11]. The size estimate from CRC is based on 2 independent captures: capture 1 and capture 2. In the first capture, participants are tagged and counted. This follows a second independent capture of participants, some of which would have been tagged in the first capture. From the proportion of participants recaptured, an estimate of the entire population is inferred [12]. For the estimate to be unbiased, CRC relies on 4 assumptions: (1) independence of samples, (2) closed population (no migration), (3) matching of individual samples in both captures, and (4) equal likelihood of capture [12].

### Objectives

Population estimates for FSWs and MSM in Uganda are particularly scarce. The World Health Organization and other multilateral organizations have made estimating the size of key populations a priority [9,13,14]. There have been challenges on quality and timely reporting, especially at the subnational level, as suggested by the same report, resulting in gaps of size estimation data. A 2-source CRC (2SCRC) has previously been conducted in Kampala for FSWs and MSM, which yielded estimates of 13,000 FSWs and 8000 MSM [15]. However, population estimates of FSWs and MSM has not been conducted outside of Kampala. To address this gap in knowledge, we utilized the 2SCRC method to estimate the size of FSWs and MSM populations in select towns in Uganda. This is the first time that CRC has been used in these areas and the first population size estimates for MSM outside of Kampala.

## Methods

### Target Population

We conducted the CRC activity among FSWs in 11 locations (Tororo, Busia, Mbarara, Mbale, Gulu, Kabale, Kabarole, Jinja, Masaka, Wakiso, and Mukono) and among MSM in 7 locations (Mukono, Wakiso, Jinja, Kabarole, Gulu, Mbale, and Mbarara). These locations were chosen based on the availability of funding and the availability of MSM and FSW community-based organizations (CBOs) in these areas. The HIV prevalence in these districts is higher than the national average. FSWs who are 15 years or older and MSM who are 18 years or older were identified by FSW and MSM peers, respectively.

### Sample Size and Precision

There were no previous estimates of MSM and FSWs in these locations. To determine the target ranges for the number of unique objects to distribute per population for each town, 0.5% of all adult females and 1.5% of all adult males living in that particular location were assumed to be FSWs and MSM, respectively [16].

### Capture-Recapture Implementation

**Figure 1:**
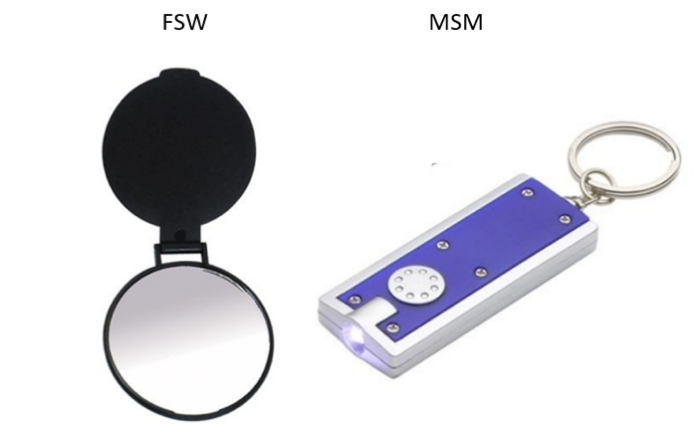
Selected unique objects by population (unique phrases not shown).

CBOs who operated in each location were consulted before the data collection to discuss the selected objects and recommend peer distributors for each target population. They provided a list of FSW and MSM peers (other members of the target population) who would act as distributors of unique objects. Objects were selected in collaboration with local CBOs and were vetted by target population members. The unique objects had unique phrases printed in various colors. Hand mirrors and keychains were distributed to FSWs and MSM, respectively, as shown in Figure 1.

In total, 2 FSW and 2 MSM peers were chosen to distribute unique objects for the first capture. Another set of 2 FSW and 2 MSM peers were assigned to collect data for the second capture. All distributors participated in a half-day training. Trainings were conducted separately for MSM and FSWs and for captures 1 and 2. The training included the purpose of the activity, a description of the target population, and instructions for data collection and documentation.

### Data Collection

Estimation was piloted first in Mbale between June 7 and 21, 2017, and then initiated in 9 phases. Phase 1, which included Jinja, Mukono, Busia, and Malaba, was conducted between June 26 and July 13, 2017. Phase 2, which included Masaka, Mbarara, Kabale, and Wakiso, was conducted between July 17 and August 4, 2017. Phase 3, which included Kabarole and Gulu, was conducted between August 7 and 25, 2017.

All data were collected using Open Data Kit Collect, an open source Android app, on a smartphone [17]. The first sample was captured by distributing unique objects by MSM and FSW peers to the target population. Distributors were asked to visit all known hotspots at different times of the day. When the distributors came in contact with a target population member, they were told to estimate the age group of the individual, indicate the Global Positioning System (GPS) coordinates of where the object was given out, and identify if the participant accepted the unique object. Distributors were also asked to randomly give out unique objects at all possible places where target population members might congregate. Per the protocol, any FSW estimated to be under the age of 18 was referred to specialized services.

The second capture took place approximately 5 to 7 days after the first capture, with a different set of peers to collect the data. No objects were distributed during capture 2, and distributors asked target population members if they had received a gift in the previous week. If they indicated that they had received a gift, they were asked to show the distributor the object. If the approached individual claimed to have received an object but did not have the object with them, they were asked to identify the correct object from a piece of paper with pictures of 10 to 15 different objects (some similar to the real objects, some very different). Distributors recorded the picture the individual identified but did not reveal whether they were correct or not. Distributors also electronically collected the same demographic and geographical data as in the first capture.

### Data Analysis

All PSE calculations were carried out using a CRC calculator developed by Simple Interactive Statistical Analysis [18]. Nonweighted data were used for analysis. Using the 2 data sources from captures 1 and 2, the CRC method provides an estimate by the following formula:

N=MC/R

where *N* is the estimate of the total population size, *M* is the total number of people captured and marked on the first visit, *C* is the total number of people captured on the second visit, and *R* is the number of people captured on the first visit and then recaptured on the second visit.

To calculate the CI to give a range of error for the estimate of total population, the following formula was used:

95%CI= N +/- 1.96 *sqrt(Var(N))

where variance is calculated as follows:

Var(N) =[ MC * (M-R)*(C-R)]/(R^3)

Size estimates were calculated using 2 different definitions: (1) those were able to present the object or identify the correct object from a set of pictures and (2) only those who were able to present the object. All values were rounded to the next whole number.

## Results

### Sampling

We show the sampling results using 2 different recapture definitions: (1) those who are able to present the object or identify the correct object from a set of pictures and (2) those who are only able to present the object. The numbers of objects offered, how many FSWs/MSM refused objects, the number of MSM/FSWs in captures 1 and 2, and the number of people captured twice (recaptures) are shown (Tables 1 and 2).

We show the number of MSM or FSWs who misidentified unique objects from a set of pictures (Table 3). We see that in some towns, no misidentification of objects occurred. We present the proportion of capture 2 that was recaptures for each distributor for each town (Tables 4 and 5).

**Table 1:** Target sample size, number of objects distributed, number of refusals, number of female sex workers (FSWs) in capture 1, number of FSWs in capture 2, number of recaptures for recapture definition 1, and number of recaptures for recapture definition 2 for FSWs in 11 Ugandan towns.

Town	Objects offered, n	Refusals, n	Capture 1, n	Capture 2, n	Recapture definition 1, n	Recapture definition 2, n
Mbale	143	32	111	156	31	25
Jinja	103	8	95	194	23	23
Busia	117	9	108	169	22	19
Tororo	66	7	59	146	16	3
Masaka	130	4	126	138	50	34
Wakiso	105	3	102	146	18	18
Mbarara	155	5	150	203	16	16
Kabale	86	21	65	110	22	19
Gulu	141	8	133	225	32	21
Kabarole	112	10	102	183	55	47
Mukono	159	9	150	242	116	113

**Table 2:** Target sample size, number of objects distributed, number of refusals, number of men who have sex with men (MSM) in capture 1, number of MSM in capture 2, number of recaptures for recapture definition 1, and number of recaptures for recapture definition 2 for MSM in 7 Ugandan towns.

Town	Objects offered, n	Refusals, n	Capture 1, n	Capture 2, n	Recapture definition 1, n	Recapture definition 2, n
Mbale	129	28	101	143	46	38
Jinja	81	0	81	95	17	7
Wakiso	81	2	79	149	62	32
Mbarara	99	0	99	120	51	37
Gulu	109	7	102	153	88	87
Kabarole	92	8	83	133	61	33
Mukono	106	2	104	142	88	56

**Table 3:** Number of female sex workers and men who have sex with men who misidentified the objects.

Town	Female sex workers who misidentified objects, n	Men who have sex with men who misidentified objects, n
Mbale	5	13
Jinja	6	0
Busia	0	—^a^
Tororo	1	—
Masaka	0	—
Wakiso	0	0
Mbarara	0	10
Kabale	0	—
Gulu	2	5
Kabarole	0	8
Mukono	2	0

^a^—: not applicable.

**Table 4:** Recaptures for distributors 1 and 2 for female sex workers per each town.

Town	Recaptures for distributor 1, n	Recaptures for distributor 1/capture 2, n	Recaptures for distributor 2, n	Recaptures for distributor 2/capture 2, n
Mbale	6	0.04	19	0.12
Jinja	7	0.04	16	0.08
Busia	7	0.04	16	0.09
Tororo	3	0.02	0	0.00
Masaka	15	0.11	19	0.14
Wakiso	1	0.01	17	0.12
Mbarara	16	0.08	0	0.00
Kabale	9	0.08	10	0.09
Gulu	10	0.04	11	0.05
Kabarole	5	0.03	42	0.23
Mukono	5	0.02	109	0.45

**Table 5:** Recaptures for distributors 1 and 2 for men who have sex with men per each town.

Town	Recaptures for distributor 1, n	Recaptures for distributor 1/capture 2, n	Recaptures for distributor 2, n	Recaptures for distributor 2/capture 2, n
Mbale	6	0.04	32	0.22
Jinja	5	0.05	2	0.02
Wakiso	21	0.14	11	0.07
Mbarara	17	0.14	20	0.17
Gulu	19	0.12	68	0.44
Kabarole	26	0.20	7	0.05
Mukono	36	0.25	20	0.14

**Table 6:** Population size estimates per town for female sex workers.

Town	Adult female population, N	PSE^a^ of recapture definition 1 , n (PSE/total Population size as % of recapture definition 1)	95% CI	PSE of recapture definition 2, n (PSE/total population size as % of recapture definition 2)	95% CI
Mbale	161,720	559 (0.35)	410-708	693 (0.43)	474-912
Jinja	144,280	802 (0.56)	534-1069	802 (0.56)	534-1069
Busia	98,210	830 (0.85)	542-1119	961 (0.98)	592-1330
Tororo	155,520	539 (0.35)	326-751	2872 (1.85)	0-6005
Masaka	91,680	348 (0.38)	288-408	512 (0.56)	384-639
Wakiso	786,970	828 (0.11)	503-1152	828 (0.11)	502-1152
Mbarara	156,270	1904 (1.22)	1058-2749	1904 (1.22)	1058-2749
Kabale	75,809	325 (0.43)	227-424	377 (0.50)	247-506
Gulu	88,820	936 (1.05)	674-1197	1425 (1.60)	893-1958
Kabarole	90,307	340 (0.38)	289-391	398 (0.44)	326-469
Mukono	194,920	313 (0.16)	294-333	322 (0.17)	300-343

^a^PSE: population size estimation.

**Table 7:** Population size estimates per town for men who have sex with men.

Town	Adult male population, N	PSE^a^ of recapture definition 1 , n (PSE/total Population size as % of recapture definition 1)	95% CI	PSE of recapture definition 2, n (PSE/total population size as % of recapture definition 2)	95% CI
Mbale	123,201	314 (0.25)	259-370	381 (0.31)	299-462
Jinja	18,233	453 (2.48)	280-626	1100 (6.03)	351-1849
Wakiso	15,228	190 (1.25)	173-207	368 (2.42)	281-455
Mbarara	48,754	233 (0.48)	200-267	322 (0.66)	253-390
Gulu	38,069	178 (0.47)	169-187	180 (0.47)	170-189
Kabarole	13,569	181 (1.33)	164-199	335 (2.47)	258-412
Mukono	40,686	168 (0.41)	160-177	264 (0.65)	228-301

^a^PSE: population size estimation.

### Population Size

We present the results of the population size estimates of FSWs and MSM, the 95% CI, the number of adult male or female population of that particular town, and the prevalence of MSM or FSWs (Tables 6 and 7).

#### Female Sex Workers

We estimated the number of FSWs in Mbale to be 693 (95% CI 474-912). For Jinja, Mukono, Busia, and Tororo, we estimated the number of FSWs to be 802 (95% CI 534-1069), 322 (95% CI 300-343), 961(95% CI 592-1330), and 2872(95% CI 0-6005), respectively. For Masaka, Mbarara, Kabale, and Wakiso, we estimated the number of FSWs to be 512 (95% CI 384-639), 1904 (95% CI 1058-2749), 377 (95% CI 247-506), and 828 (95% CI 502-1152), respectively. For Kabarole and Gulu, we estimated the number of FSWs to be 397 (95% CI 325-469) and 1425 (95% CI 893-1958), respectively.

#### Men Who Have Sex With Men

We estimated the number of MSM in Mbale to be 381 (95% CI 299-462). For Jinja and Mukono, we estimated the number of MSM to be 1100 (95% CI 351-1849) and 264 (95% CI 228-301), respectively. For Mbarara and Wakiso, we estimated the number of MSM to be 322 (95% CI 253-390) and 368 (95% CI 281-455), respectively. For Kabarole and Gulu, we estimated the number of MSM to be 335 (95% CI 258-412) and 180 (95% CI 170-189), respectively.

## Discussion

### Principal Findings

We conducted multiple size estimation activities using the CRC method and present 2 size estimates for FSWs and MSM in 11 select Ugandan towns.

Comparing the population estimates from the different recapture definitions for FSWs and MSM, we see that recaptures of those who had the object or were able to identify the object were generally higher, resulting in lower population estimate compared with the results from those who just had the object with them.

Our subnational estimates for FSWs as percentage of the adult female population, or FSWs prevalence, range from 0.3% to 1.2%. In Tororo, we found that women were less likely to keep the object with them. This is evident from a comparison of the number of recaptures in Table 1. Only 3 women had the object with them, but an additional 13 women were able to accurately identify the object from a set of pictures. Perhaps, this is because the women lost the objects more frequently or were selling the objects, which may reflect the socioeconomic status of women in Tororo. When women who could identify the object from a set of pictures were included in the analysis, the size estimate dropped from 2872 (FSWs prevalence of 1.85%) to 539 (FSWs prevalence of 0.35%). Our range of FSW prevalence is similar to subnational FSWs prevalence estimates in other regions of Africa (0.1%-12.0%) [19]. Generally, in other areas, the results did not differ as much because the majority of FSWs kept the object for a week. Our subnational estimates for MSM as percentage of the adult male population, or MSM prevalence, range from 0.03% to 0.9%. When compared with global estimates of MSM (3%-5%), we find that most of our MSM prevalence estimates are lower [20,21]. These results reflect the difficulties in executing venue-based population estimates in small settings in countries such as Uganda and may suggest that a large proportion of MSM was not recognizable to distributors or that they do not frequent venues that distributors targeted, or that these smaller towns indeed harbor fewer MSM.

We can compare our FSWs and MSM estimates with results from a 2014 report by the Ugandan Ministry of Health and Uganda AIDS Commission where they estimated 54,549 sex workers (0.57% of the adult female population) and 10,533 MSM (0.12% of the adult male population) in Uganda [15]. With the exception of our FSWs prevalence estimates from Mbale, our FSW and MSM prevalence estimates are higher compared with the FSWs and MSM prevalence found in the report. However, it is important to note that the report estimates are national, whereas our estimates are subnational. Unfortunately, because of lack of data in the literature, we were not able to compare our subnational estimates to other population estimates.

The Priorities for Local AIDS Control Effort (PLACE) method has been utilized for FSWs in some of these locations [22]. Though the PLACE report did not provide population size estimates for FSWs, we can compare the numbers of FSWs reported using the PLACE method. Per PLACE method, there were 65 FSWs in Jinja, 62 FSWs in Mbale, 61 FSWs in Kabale, 70 FSWs in Masaka, 119 FSWs in Malaba, and 113 FSWs in Mbarara. The numbers of FSWs found using PLACE are lower than the number of FSWs captures for each corresponding location. This is likely explained by the PLACE methodology, where only 3 areas within the district are visited, whereas our distributors visited multiple hotspots.

### Strengths and Limitations

There were a number of limitations in our PSE activity that is inherent to CRC methodology and failing to meet its assumptions. As we did not want to collect personal identifying information, we used unique objects to identify recaptures. However, not every person carried the unique object with them, which made determining the exact number of recaptures problematic because we could not confirm that the person who received the object was the same person who received it. Estimates would thus be too high if matches were not identified or too low if recaptures were matched incorrectly. We attempted to reduce the bias from using unique objects as a matching mechanism by instructing participants to keep the object with them for as long as the data collection period (5 to 7 days) and limiting the time between captures to 5 to 7 days. However, there were individuals from both populations who did not have the object with them. This is not surprising, given that a population member may have lost the object, given it away, or left it at home. Minimizing the time between captures was also an effort to meet the assumption of a closed population (no in and out migration). If there is an increase in the number of people in the second sample because of migration, it may result in an underestimate, or vice versa. In addition, we tried to meet the assumption of equal likelihood of capture by instructing the peer distributors to visit all known hotspots and randomly sample individuals. In certain towns, this may not have been possible, and our peer distributors may not have been able to visit all hotspots in the allotted time. Our method involved approaching population members at select hotspots and may have excluded specific subgroups that may not attend venues (eg, street- or home-based FSWs or MSM/FSWs who meet individuals or clients using social media or other app). Individuals with higher social visibility are more likely to be captured; thus, our results are likely to be underestimated for all populations. The expansion of captures to various other data sources (not just object distribution) to include service lists and or social media and websites could reach those individuals who are less likely to attend venues. Furthermore, members of the FSWs and MSM populations may have decided to not participate in the activity for fear of being identified. The use of FSW and MSM peers for data collection may have alleviated this issue. Finally, we attempted to meet the assumption of independence of samples by using different staff for captures 1 and 2.

We encountered unique observations and challenges during our size estimation activities. First, the PSE activity was well received by the community with the help of the local CBO. The unique objects were generally well liked by the target population members. In Mukono, the unique objects were too well liked; FSW distributors reportedly were selling objects, and thus the activity was repeated with a new set of peers. Tainted data from Mukono were discarded, and the Mukono data presented relied on the repeated activity. We also found that in some areas, target population members would surround distributors hoping to receive an object, suggesting that objects may not have always been given out at random and that not everyone had an equal chance of being captured. To ensure that these issues do not occur in the future, we recommend consultation with local CBOs and to choose unique objects that are likeable but not overly popular. Furthermore, although we instructed data collectors to go to any place where they might find a member of the target population within the district, the majority of the captures were found in the town centers. In addition, considering that each distributor had the same training and used the same electronic data collection tool, we expected that the proportion of captures that were recaptures be similar between the 2 FSWs or MSM distributors for capture 2. However, we saw that in many sites, 1 distributor had substantially more (>10%) recaptures than the other. We attempted to mitigate this issue by looking at the GPS data for each of the distributor’s captures, to see if they made up certain captures. However, it was difficult to assess which captures were true captures. To the extent that we can check, we did not find any fabricated captures. We also checked to see if the location of the recaptures were in similar locations to those of captures 1 and 2 and found that they were, suggesting that the recaptures were recorded in locations where FSWs and MSM congregate and are thereby legitimate. Regardless, these issues brought to light that one of the most critical elements of a successful CRC activity using unique objects is choosing the right distributors.

### Conclusions

New empirical population size estimates were generated for MSM and FSWs in select towns in Uganda. These estimates are among the first in these locations in Uganda and are important in providing knowledge and insight in planning for HIV programs by stakeholders working in Uganda. More implementation research and more systematic use of CRC or other empirical PSE methods are warranted for Uganda. PSE activities should be included in future HIV surveillance efforts to improve estimates and optimally plan for the provision of services to high-risk populations.

## Abbreviations

CBOs: community-based organizations
CRC: capture-recapture
FSWs: female sex workers
GPS: Global Positioning System
MSM: men who have sex with men
PLACE: Priorities for Local AIDS Control Effort
PSE: population size estimation
